# The use of systemic immune inflammatory index as a predictor for nematodes infections in horses

**DOI:** 10.1016/j.parepi.2025.e00453

**Published:** 2025-07-31

**Authors:** Falmata Kyari, Cephas Joseph Pogu, Ismaila Alhaji Mairiga, Lawan Adamu

**Affiliations:** a Faculty of Veterinary Medicine, Department of Veterinary Parasitology, University of Maiduguri, Maiduguri P.M.B. 1069, Borno State, Nigeria; b Faculty of Veterinary Medicine, Department of Veterinary Medicine, University of Maiduguri, Maiduguri P.M.B. 1069, Borno State, Nigeria

**Keywords:** Systemic immune inflammatory index, Nematode, Horses, Platelets, Leucocytes, Neutrophils

## Abstract

**Background:**

Nematode infections are a significant health concern in horses, causing a range of clinical signs and economic losses. Early detection and diagnosis are crucial for effective treatment and management.

**Objectives:**

Examining the application of the systemic immune Inflammatory index (SII) as a predictor for nematode infections in horses, using platelets count, leucocytes count, and neutrophils count.

**Methods:**

A cross-sectional study was conducted on 164 horses, consisting of 66 horses with nematode infections and 98 horses without infections. The SII was computed using the platelets count, leucocytes count, and neutrophils count. Receiver operating characteristic (ROC) curve analysis was used to evaluate the SII's diagnostic accuracy.

**Results:**

Nematode infections were severe in horses with mixed infections, with an average of 1805.90 ± 292.68 eggs per gram (epg). Notably, among specific species, *Cyathostomum* spp., exhibited a significantly different average of 2264.29 ± 132.61epg compared to other nematodes. There is a significant negative correlations between the systemic immune-inflammatory index (SII) and the Eggs per gram count for nematodes infections at (*r* = −0.6023; *P* < .0001). The SII values were significantly lower (0.06) in horses with nematode infections compared to those without infections (0.19) at *p* < .001. With an area under the ROC curve (AUC) of 0.990, the SII demonstrated exceptional diagnostic precision. For the SII, the ideal cut-off value is ≤0.108, with a sensitivity of 98.5 % and a specificity of 100 %. The ROC curve was validated using the Youden index (J) with a higher value of 0.9848 indicating better performance.

**Conclusion:**

The study demonstrated that the SII is a reliable predictor for nematode infections in horses, using platelets count, leucocytes count, and neutrophils count. The SII is a non-invasive, reasonably priced method for identifying and diagnosing nematode infections in horses.

## 1 Introduction

Nematode infections are a pervasive and debilitating issue in equine health, causing significant morbidity and mortality worldwide particularly in young, old, or immunocompromised animals (Burke, 2024). Nematode parasites, such as *Cyathostomum* spp., *Oxyuris* spp., *Strongylus* spp., and *Parascaris* spp., are ubiquitous in equine populations' worldwide (Burke, 2024; Matthews et al., 2023; Yehia et al., 2024; Nielsen and Reinemeyer, 2018; Scare et al., 2018). Clinical signs of nematode infections in horses can range from mild to severe and include weight loss, diarrhea, colic, and respiratory distress (Matthay et al., 2019). Early detection and treatment are crucial to prevent serious health consequences (Verocai et al., 2020). However, current diagnostic methods, including fecal egg counts and coproculture, have limitations in terms of sensitivity and specificity (Verocai et al., 2020). Horses at danger of nematode infections can now be identified using the Systemic Immune Inflammatory Index (SII), a promising biomarker (Oflar et al., 2024; Krofič Žel et al., 2024; Germolec et al., 2018; Ye et al., 2022; Saylik and Akbulut, 2022).

The SII reflects the systemic inflammatory response to nematode infections, offering a non-invasive and cost-effective tool for predicting infections (Vinther et al., 2016). This index is calculated using routine hematological parameters, including platelets count (Middleton and Rondina, 2016; Hayward, 2018; George and Aster, 2009; Cooper and Ghanima, 2019), leucocytes count (Russell et al., 2019; Patel et al., 2021), and neutrophils count (Dale et al., 2008; Cassatella et al., 2019). The SII is a novel biomarker that has been proposed as a predictor for nematode infections in horses (Nielsen and Reinemeyer, 2018; Russell et al., 2019). The following formula is used for computing this index:

$$\mathrm{SII}=\left(\text{Platelet count}\;x\;\text{Neutrophil count}\right)/\text{Leucocyte count}$$

The SII has been shown to be a reliable predictor of hepatic alveolar Echinococcosis in human patients, with elevated SII values indicating increased systemic inflammatory immune response and patients with high SII had a noticeably shorter survival period (Ren et al., 2021). The calculation of SII using platelets count (Schafer et al., 2003), leucocytes count, and neutrophils count is a simple and cost-effective method (Amulic et al., 2012). Platelets count is an indicator of thrombocytosis, which is often seen in response to Inflammatory and infection (Faurschou and Borregaard, 2003). Leucocytes count is an indicator of the overall immune response, with elevated counts indicating Inflammatory or infection (Bodis et al., 2018). Neutrophils count is an indicator of the acute inflammatory response, with elevated counts indicating bacterial or parasitic infections (Faurschou and Borregaard, 2003; Foster and Elsheikha, 2012; Schattner et al., 2019).

Equids generally display a tolerant immune response to nematodes rather than an aggressive one (Tombak and Rubenstein, 2023). This means that instead of launching a strong inflammatory reaction, horses may focus on tissue repair and maintaining their body condition, even in the presence of a chronic infection (Reis et al., 2024). Additionally, studies have shown that gastrointestinal nematode infections in horses can lead to haemo-biochemical alterations, including changes in neutrophil and leukocyte counts (González-Garduño et al., 2021). However, these changes are not necessarily exclusive to nematode infections other conditions, such as bacterial infections, stress, or metabolic disorders, could also influence the SII (Khan and Collins, 2004).

In human patients with stable angina pectoris receiving percutaneous coronary intervention (PCI), prior studies demonstrated a substantial correlation between SII and coronary artery disease (CAD) severity, high Syntax score (SxS), and major adverse cardiovascular and cerebrovascular events (MACCE) (Karakayali et al., 2024). Moreover, a high SII score has no relationship to a poor clinical prognosis, and SII has been demonstrated to predict both in-hospital and long-term clinical outcomes for elderly acute myocardial infarction (AMI) patients who get PCI (Karakayali et al., 2024). Hence, an independent correlation between the presence of ischemia with the non-obstructive coronary artery (INOCA) and a high SII level was discovered (Karakayali et al., 2024). Numerous studies in human patients have shown a correlation between hypertension and inflammatory indicators, such as interleukin-6, C-reactive protein, and white blood cell counts (Karakayali et al., 2023). In human patients, variability in blood pressure is similarly linked to inflammatory indicators (Karakayali et al., 2023). According to Karakayali et al.'s study, patients with reverse-dipper hypertension had a higher SII level than those with dipper or non-dipper hypertension (Karakayali et al., 2023). Therefore, the current research aims to explore the current evidence on the use of SII as a predictor for nematode infections in horses, discussing its potential applications, and advantages in equine clinical practice.

## 2 Materials and methods

### 2.1 Study design

This study aims to investigate the use of the systemic immune Inflammatory index (SII) as a predictor for nematode infections in horses. The study was conducted in Maiduguri (11${}^{{}^{\circ}}$ 50′ N 13${}^{{}^{\circ}}$ 09′ E. It occupies an area of 50,778 km^2^).

A cross-sectional design was employed, where horses with and without nematode infections were recruited and their SII values calculated and compared. At 8:30 AM, blood and fecal samples were collected from the recruited horses. The horses, which appeared to be in good health, underwent a thorough clinical examination to evaluate their current health status. The sampling process was conducted carefully to ensure that the horses were not stressed by the procedure, which could have disrupted the immunologic and hematological parameters.

### 2.2 Animals

Blood and fecal samples were collected from a total of 164 horses for the current study in Maiduguri, Borno State. The horses were divided into two groups: Infected group (*n* = 66): Horses with confirmed nematode infections (*Cyathostomum* spp., = 7 horses, *Oxyuris* spp., = 15 horses, *Strongylus* spp. = 13 horses and *Parascaris* spp. = 14 horses) and those with mixed infections (*Oxyuris* spp.*, Parascaris* spp.*, Strongylus* spp.*, Cyathostomum* spp. = 7 horses, while those with *Parascaris spp, Strongylus spp, Oxyuris* spp. = 10 horses) were recruited for this group. Non-infected group (*n* = 98): Horses without nematode infections were recruited for this group.

### 2.3 Inclusion criteria

Horses were included in the study with the following criteria:

Age: 1–20 years, Breed: Any breed, Sex: Male or female, Weight: 300–600 kg and Health status: Apparently horses with no underlying health issues.

### 2.4 Exclusion criteria

Horses were excluded from the study if they meet any of the following criteria: Age: <1 year or > 20 years, Weight: <300 kg or > 600 kg, Health status: Horses with underlying health issues (e.g., laminitis, colic, bacterial infections, trauma, co-infections, or inflammatory diseases), and Medication: Horses receiving anti-parasitic medication or immunosuppressive therapy.

### 2.5 Sample collection

The horses were properly restrained and an antiseptic gauze was used to disinfect the site of collection by swiping, this helps remove superficial dirt as well help in visualizing the raised vein after which a sterile needle was used to collect 4mls of blood from the jugular vein, the needle was removed gently and the collected blood is then transferred into a well labelled EDTA tubes and submitted to the laboratory for analysis.

### 2.6 Laboratory analysis

The following hematological parameters were measured using (Hettich-Hematocrit 210 and Hawksley microhematocrit reader):

Platelets count (PLT), Leucocytes count (WBC), and Neutrophils count (NEUT) The SII was calculated using the following formula:

$$\mathrm{SII}=\frac{\left(\mathrm{PLT}\;x\;\mathrm{NEUT}\right)\;}{\mathrm{WBC}.}$$

### 2.7 Parasitological examination

Fecal samples were collected from each horse and submitted to the laboratory for parasitological examination at the University of Maiduguri's faculty of veterinary medicine, immediately from each horse's rectal ampulla using disposable arm-length gloves and kept in a universal bottle until analysis as previously reported by Hendrix and Robinson. The nematode eggs were identified using the simple flotation test based on their size, shape, color, content (embryo/larvae), and lack of operculum (Scare et al., 2018; Hendrix and Robinson, 2022), Nielsen et al. (Nielsen et al., 2006) and Kaplan and Nielsen (Kaplan and Nielsen, 2010) used the McMaster method (Vysniauskas et al., 2004) to calculate the number of eggs per gram of feces (EPG). As a result, the Equidae are classified as mild if their egg count level falls between 0 and 799 EPG, moderate if it falls between 800 and 1200 EPG, and severe if it exceeds 1200 EPG (Taylor et al., 2015; Soulsby, 1982).

### 2.8 Statistical analysis

The data were analyzed using statistical software (MedCalc® Statistical Software version 23.0.9 MedCalc Software Ltd., Ostend, Belgium; https://www.medcalc.org; 2024). A *P*-value of less than 0.05 was used to determine that an analysis was significant. The SII values were compared between the infected and non-infected groups using the independent samples *t*-test. One way ANOVA was used to compare the EPG between the specific nematodes species, Tukey Kramer was used as post hoc test to compare the groups. Receiver operating characteristic (ROC) curve analysis was performed using R Studio to evaluate the diagnostic accuracy of the SII and the validation of the ROC curve Results was performed using Youden index.

## 3 Results

The present study aimed to investigate the use of the systemic immune Inflammatory index (SII) as a predictor for nematode infections in horses. The SII was calculated using the platelets count, leucocytes count, and neutrophils count.

**Fig. 1:**
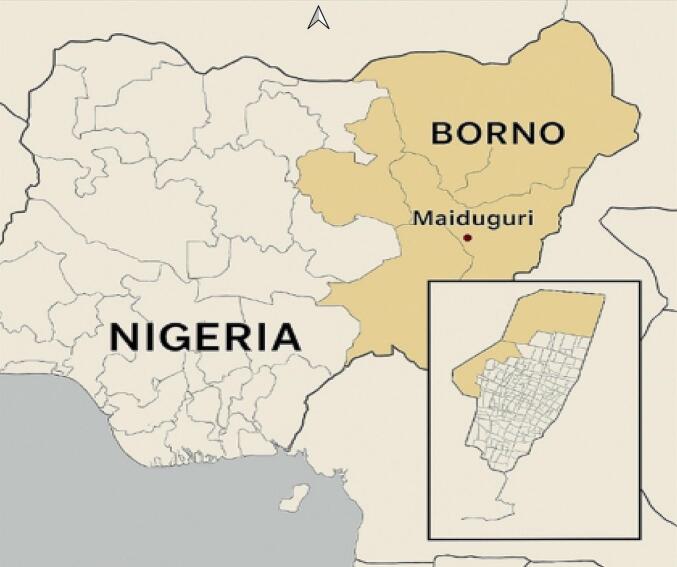
Map of Nigeria showing Maiduguri, Borno State. (Source: QGIS; https://qgis.org).

**Fig. 2:**
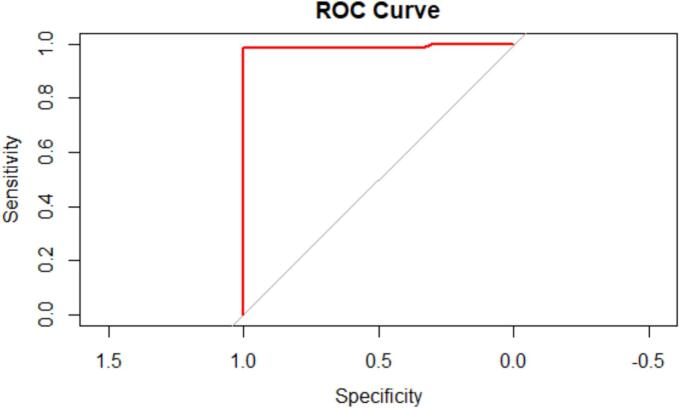
ROC curve for SII in predicting nematode infections in horses, sensitivity: 98.5; specificity: 100.0; AUC = 0.990 and the criterion value: ≤ 0.108.

The descriptive statistics for the positive platelets count (116.00 ± 44.03 × 10^3^/μL), leucocytes count (10.19 ± 3.96 × 10^9^/μL) neutrophils count (6766.02 ± 3483.94 μL), and SII values (0.06 ± 0.03) are presented in Table 1. While, Table 2, Table 3 presents the specific and mixed nematodes infections in horses, showing severe infections (1805.90 ± 292.68 epg) in the mixed infected group of the horses at *P* < .05 compared to the non-infested group. Fig. 1, showed the map of the study area. Fig. 2 presents the ROC curve portraying the accuracy and the reliability of the diagnostic test. The ANOVA analysis revealed an F-statistic of 8.46 (F-test = 8.46; *P* < .0001). The F-test evaluates whether there are statistically significant differences between group means. A high F-statistic paired with a low *p*-value (< 0.05) indicates that at least one group differs significantly, suggesting a meaningful effect size. Table 4 displayed significant negative correlations between the systemic immune-inflammatory index (SII) and the Eggs per gram count for nematodes infections at (*r* = −0.6023; P < .0001). In the current study, the prevalence of specific and mixed nematode species infections in horses were as follows, *Cyathostomum* spp. (4.27 %), *Oxyuris* spp. (9.15 %), *Strongylus* spp. (7.93 %), *Parascaris* spp. (8.54 %), (*Oxyuris* spp., *Parascaris* spp., *Strongylus* spp., and *Cyathostomum* spp. 4.27 %), and (*Parascaris* spp., *Strongylus* spp., and *Oxyuris* spp. 6.09 %). Table 5 depicts the area under the curve (AUC) while Table 6 showed the validation of the ROC curve Results using Youden index.

**Table 1:** Status of Nematodes Infections using Platelets count, Leucocytes count, Neutrophils count and SII Values.

Status of Nematodes Infections	n	Platelets(X10^3^/μL)	Leucocytes(10^9^/μL)	Neutrophils(μL)	SII Value
Positive (P)	66	116.00^b^ ± 44.03	10.19^a^ ± 3.96	6766.02^a^ ± 3483.94	0.06^b^ ± 0.03
Negative (N)	98	295.93^a^ ± 79.09	9.74^a^ ± 1.15	6424.59^a^ ± 895.29	0.19^a^ ± 0.06

All values are means ± SD. ^a, b^ within each column, means with superscripts are significantly different at *P* < .05.

**Table 2:** Specific Nematodes species Infections in horses.

Specific Nematodes species Infections	n	EPG (Egg per gram)
*Cyathostomum* spp.	7	2264.29^a^± 132.61
*Oxyuris* spp.	15	470.67^b^± 62.47
*Parascaris* spp.	14	1640.00^a^±239.38
*Strongylus* spp	13	1515.38^a^± 178.23

All values are means ± SE. ^a b,^ within each column, means with superscripts are significantly different at *P* < .05.

**Table 3:** Mixed Nematodes species Infections in horses.

Mixed Nematodes species Infections	n	EPG (Egg per gram)
*Oxyuris* spp.*, Parascaris* spp.*, Strongylus* spp.*, Cyathostomum* spp	7	1382.86^ab^± 339.93
*Parascaris* spp.*, Strongylus spp, Oxyuris spp*	10	1805.90^a^± 292.68

All values are means ± SE. ^a, b,^ within each column, means with superscripts are significantly different at *P* < .05.

**Table 4:** Correlation between Systemic Immune Inflammatory Index (SII) and Eggs per gram count in nematodes infections.

Variables	Coefficient of correlation (r)	95 % C·I (Lower & Upper limits		P-Value
SII By EPG	−0.6023	−0.6917	−0.4947	< 0.0001

SII = Systemic Immune Inflammatory Index, EPG = Eggs per grams count.

**Table 5:** Area under the ROC curve (AUC).

Area under the ROC curve (AUC)	0.990
Standard Error ^a^	0.0103
95 % Confidence interval ^b^	0.960 to 0.999
z statistic	47.763
Significance level P (Area = 0.5)	**<0.0001**

**Table 6:** Validation of the ROC curve Results using Youden index.

**Youden index J**	0.9848
**Associated criterion**	≤0.108
**Sensitivity**	98.48
**Specificity**	100.00

The preceding equation was used to determine the SII:

$$\mathrm{SII}=\frac{\left(\mathrm{Platelets count}\;x\;\mathrm{Neutrophils count}\right)\;}{\mathrm{Leucocytes count}\;\left(\mathrm{WBC}\right)\;}$$

Receiver Operating Characteristic (ROC) Curve Analysis.

The ROC curve was plotted using the SII value against the status of nematodes (both positives and negatives values). At various cut-off levels for the SII, the ROC curve displayed the correlation between the true positive rate (sensitivity) and the false positive rate (100-specificity). The area under the curve (AUC) represents the diagnostic accuracy of the SII. The ROC curve analysis was performed to evaluate the diagnostic accuracy of the SII in predicting nematode infections in horses. The ROC curve is presented in Fig. 2. The area under the ROC curve (AUC) was 0.990, indicating excellent diagnostic accuracy. The sensitivity and specificity of the SII in predicting nematode infections were 98.5 % and 100 %, respectively.

### 3.1 Cut-off value

The optimal cut-off value for the SII was determined to be ≤0.108, which corresponded to a sensitivity of 98.5 % and a specificity of 100 %. The study demonstrated that the SII, calculated using the platelets count, leucocytes count, and neutrophils count, is a reliable predictor for nematode infections in horses. The SII showed excellent diagnostic accuracy, with an AUC of 0.990. The optimal cut-off value for the SII was determined to be ≤0.108. According to these results, the SII might be a useful diagnostic tool for determining whether horses are susceptible to nematode infections.

### 3.2 Youden index

The Youden index (J) assesses the performance of a diagnostic test at a specified cutoff, with a higher value indicating better performance (Parsaeian et al., 2024). This index is particularly useful when sensitivity and specificity are deemed equally important according to the diagnostic criteria (Šimundić, 2009). Under these conditions, an ideal cutoff (c) is defined by the value of J (Hassanzad and Hajian-Tilaki, 2024).

$$J={\mathrm{Max}}_{c}\;\left({\text{Sensitivity}}_{c}+{\text{specificity}}_{c}–100\right)$$

The Youden index ranges from 0 to 1, where a value of 1 indicates a perfect test and a value of 0 suggests no diagnostic value (Samawi et al., 2017). When sensitivity equals 100 - specificity, the diagonal line on the ROC (Receiver Operating Characteristic) diagram represents the minimum performance level (Carter et al., 2016). The Youden index (J-index) for that specific cutoff is determined by measuring the vertical distance between the diagonal line and the ROC curve (Martínez Barbero et al., 2025). The ROC curve itself visually represents the J-index (Martínez Barbero et al., 2025).

The best outcome occurs when both sensitivity and specificity are equal to 1, or when the false positive rate (which is calculated as 100 - specificity) is zero (Fabricant, 2024). This ideal combination is represented by the point in the upper left corner of the graph. Since sensitivity and specificity are equally important for diagnosis, the closer a ROC curve is to this ideal state, the better the diagnostic marker performs (Hassanzad and Hajian-Tilaki, 2024). The Youden index can also be represented in this same way (Hassanzad and Hajian-Tilaki, 2024).

## 4 Discussion

Nematode infections significantly affect the health and productivity of working horses, reducing their output and, as a result, diminishing both individual and regional income (Debere et al., 2018). The Use of Systemic Immune Inflammatory Index as a Predictor for Nematodes Infections in Horses using platelets count, leucocytes count, and neutrophils count has provided significant insights into the utility of the systemic immune Inflammatory index (SII) as a predictive tool for nematode infections in horses in the present study.

In the current study, the overall prevalence of nematode parasites in horses was 40.2 %. This is significantly lower than the 94.5 % prevalence reported in a previous study (Mathewos et al., 2022). Despite this lower rate, the SII successfully detected the presence of these parasites. Furthermore, in the present study, a strong and significant negative correlation between the SII and eggs per gram clearly demonstrates the diagnostic method's ability to predict which horses are harboring parasites and which are not. In a study conducted by (Candemir et al., 2021), they found a positive correlation between SII values and the Severity of Stable Coronary Artery Disease. Additionally, only in the group with severe fibrosis did SII exhibit a statistically significant negative connection with liver stiffness measurement (LSM) in another investigation (Xie et al., 2022). As the value of SII decreases, the eggs per gram value increases, indicating the presence of these parasites. Conversely, if SII increases, the eggs per gram value reduces to a minimum. The current study revealed that horses with nematode infections experienced severe health issues compared to those without infections. These findings align with the results of a study conducted by (Mathewos et al., 2022). This similarity may be attributed to the method used to accurately identify the horses harboring the nematode parasites.

Nematode infections in horses trigger a complex immune response, involving the activation of various immune cells, including neutrophils, eosinophils, and lymphocytes (Foster and Elsheikha, 2012). This finding is similar to the findings of the present study where the neutrophils, and lymphocytes were stimulated, and this could lead to the production of pro-inflammatory cytokines, such as TNF-α and IL- 1β, which promote the recruitment of immune cells to the site of infection (Abo-Aziza et al., 2017). Equids often demonstrate a tolerant immunological response to nematodes rather than an aggressive one. This implies that even when a chronic infection is present, horses may prioritize tissue healing and preserving their physical state rather than initiating a severe inflammatory response. These findings were in accord with the studies conducted by (Tombak and Rubenstein, 2023; Reis et al., 2024). This may help to explain why, unlike in other species, nematode infections in horses may not be directly correlated with the systemic inflammatory index (SII), a frequently used inflammatory marker.

In a study of the humans patients conducted by (Karakayali et al., 2024) indicated an independent correlation between the presence of ischemia with the non-obstructive coronary artery (INOCA) and a high SII level was discovered, while, another study reported on patients with reverse-dipper hypertension had a higher SII level than those with dipper or non-dipper hypertension (Karakayali et al., 2023). The lower SII values in infected horses, despite its increase in humans facing inflammatory conditions, suggests a fundamental difference in how equine immune systems regulate inflammation when dealing with nematodes infections. This discrepancy could be attributed to varying immunopathological mechanisms, possibly related to differences in innate immunity, regulatory pathways, or adaptive immune responses between humans and horses.

One possibility is that horses exhibit a more suppressive or controlled immune response to chronic nematode infections, minimizing excessive inflammation to avoid tissue damage. Unlike humans, whose immune systems often launch aggressive inflammatory responses against pathogens, horses might engage alternative pathways that dampen inflammatory markers like platelet, neutrophil, and lymphocyte ratios, resulting in a lower SII values.

The SII is a novel index that provide a comprehensive assessment of the systemic immune response (Tomita et al., 2018). In the current study, horses in the positive group with low platelet counts may indicate a severe nematode infestation, which can lead to thrombocytopenia. A low platelet count might also suggest immunosuppression, meaning the horse's immune system is weakened, making it more vulnerable to nematode infections. Furthermore, a low platelet count could be associated with co-infections, where the horse is simultaneously infected with other pathogens in addition to nematodes. However, (Cox et al., 2021; Islam et al., 2024; Lkhagvasuren, 2017) showed that Inflammatory and immune activation occurred in various diseases, including cancer and other infectious diseases using SII (Tomita et al., 2018; Mangalesh et al., 2023; Schlesinger, 2018).

The current study demonstrated that the SII is a significant predictor of nematode infections in horses. The SII values were significantly lower in horses with nematode infections compared to those without infections. This may stem from an inflammatory response to parasitic burden caused by elevated EPG in horses, potentially overwhelming their immune system or diminishing its efficacy, thereby leading to a lower SII values. Moreover, it may stem from the effects of chronic parasitism resulting from prolonged exposure to parasites, which could inhibit immune function, diminish inflammatory markers, and consequently lower the SII values. These findings were in contrast to the study of (Ye et al., 2022), which indicated that SII may be a potential biomarker for cardiovascular disease (CVD) development and elevated SII value is associated with an increased risk of CVD.

In the current study, the area under the ROC curve (AUC) showed a high value indicating the ability of SII in depicting early nematodes infections in horses, indicating its excellent diagnostic accuracy.

Similarly, a study by (Liu et al., 2025) demonstrated that the ROC curve was used as a diagnostic tool for the assessment of early detection and prognosis of gallbladder cancer (GBC).

The mechanism by which the SII predicts nematode infections in horses is likely related to the immune response to the parasites. A study by (Foster and Elsheikha, 2012) stated that the SII reflects the systemic immune activation and Inflammation, which are characteristic of the immune response to nematode infections. Additionally, a study conducted by (González-Garduño et al., 2021) revealed that horses infected with gastrointestinal nematodes affected the counts of leukocytes, neutrophils, and lymphocytes but did not impact eosinophil levels. This finding aligns with the current study's results, which showed a lower SII value due to changes in immune cells, including platelet, leukocyte, and neutrophil counts. The study has significant clinical implications for the diagnosis and management of nematode infections in horses. The SII can be used as a non-invasive and cost- effective tool for predicting nematode infections in horses.

## 5 Conclusion

In conclusion, this study has shown that the Systemic Inflammatory Index (SII) is a significant predictor of nematode infections in horses. Furthermore, the study offers a new perspective on equine immunopathology, enhancing our understanding of inflammation in non-human species. By demonstrating that the SII is a reliable and highly accurate biomarker for nematode infections, the study provides veterinarians with a valuable diagnostic tool that has practical applications in veterinary medicine.

Additionally, the discrepancies observed when comparing these findings to human studies suggest the need for further investigation into species-specific immune adaptations. This could potentially open new research avenues in comparative immunology. With this information, veterinarians can better identify horses that are susceptible to nematode infections and implement targeted treatment strategies.

## CRediT authorship contribution statement

**Falmata Kyari:** Supervision, Formal analysis. **Cephas Joseph Pogu:** Writing – original draft. **Ismaila Alhaji Mairiga:** Supervision, Investigation. **Lawan Adamu:** Writing – review & editing, Supervision, Conceptualization.

## Ethical statement

The Institutional Animal Care and Use Committee (IACUC) of the University of Maiduguri authorized the study protocol prior to its start, ensuring that it adhered to the standards of studies involving animals.

## Funding

This research received no grant from any funding agency.

## Declaration of competing interest

No conflicting interest to declare.
